# On the Origins and Dissemination of Domesticated Sorghum and Pearl Millet across Africa and into India: a View from the Butana Group of the Far Eastern Sahel

**DOI:** 10.1007/s10437-018-9314-2

**Published:** 2018-11-10

**Authors:** Frank Winchell, Michael Brass, Andrea Manzo, Alemseged Beldados, Valentina Perna, Charlene Murphy, Chris Stevens, Dorian Q. Fuller

**Affiliations:** 1 Independent Researcher, Arlington, USA; 2 Institute of Archaeology, University College London, London, UK; 3 Department of Asian, African and Mediterranean Studies, University of Naples “L’Orientale”, Piazza S. Domenico Maggiore 12, 80134 Naples, Italy; 4 Department of Archaeology and Heritage Management, Addis Ababa University, P.O. Box-1176, Addis Ababa, Ethiopia

**Keywords:** Origins of agriculture, *Sorghum bicolor*, *Pennisetum glaucum*, Nubia, Archaeobotany, Butana Group

## Abstract

Four decades have passed since Harlan and Stemler (1976) proposed the eastern Sahelian zone as the most likely center of *Sorghum bicolor* domestication. Recently, new data on seed impressions on Butana Group pottery, from the fourth millennium BC in the southern Atbai region of the far eastern Sahelian Belt in Africa, show evidence for cultivation activities of sorghum displaying some domestication traits. *Pennisetum glaucum* may have been undergoing domestication shortly thereafter in the western Sahel, as finds of fully domesticated pearl millet are present in southeastern Mali by the second half of the third millennium BC, and present in eastern Sudan by the early second millennium BC. The dispersal of the latter to India took less than 1000 years according to present data. Here, we review the middle Holocene Sudanese archaeological data for the first time, to situate the origins and spread of these two native summer rainfall cereals in what is proposed to be their eastern Sahelian Sudan gateway to the Red Sea and the Indian Ocean trade.

## Résumé

Quatre décennies se sont écoulées depuis que Harlan et Stemler ont proposé la zone sahélienne orientale comme le centre le plus probable de la domestication du sorgho bicolore. Récemment, de nouvelles données sur les impressions de semences sur les poteries du groupe Butana du IVe millénaire avant JC dans la région sud d’Atbai dans la ceinture sahélienne d’Afrique orientale montrent des preuves d’activités de culture du sorgho présentant certains traits de domestication. *Pennisetum glaucum* pourrait être en cours de domestication peu après dans l’ouest du Sahel, puisque le millet perlé entièrement domestiqué est. présent dans le sud-est du Mali vers la seconde moitié du troisième millénaire avant J.-C. et présent dans l’est du Soudan au début du deuxième millénaire avant notre ère. La dispersion de ce dernier en Inde a pris moins de mille ans selon les données actuelles. Nous examinons ici pour la première fois les données archéologiques soudanaises de l’Holocène moyen pour situer les origines et la propagation de ces deux céréales de pluie estivales indigènes dans ce qui est. proposé comme leur porte d’entrée soudanienne du Sahel oriental à la mer Rouge et à l’océan Indien.

## Introduction

Sorghum (*Sorghum bicolor*) and pearl millet (*Pennisetum glaucum*) formed an integral part of the caloric base of most Neolithic and Iron Age food-producing societies in the Sahelian belt (Bourlag 1996; Harlan 1992) and, beyond the scope of this paper, elsewhere in sub-Saharan Africa. In light of new evidence that has begun to place the domestication processes of both cereals in time and space, we re-assess the early evidence of these crops within the context of the mid-Holocene and Neolithic cultural traditions of Sahelian Sudan, which we model as having played key roles in sorghum domestication and pearl millet dispersal. The aims of this paper are twofold: first to provide the regional archaeological context in which sorghum domestication can be framed, and second, to consider the eastern Sudan as the gateway through which both domesticated sorghum and pearl millet passed en route to the Red Sea and Indian Ocean trade networks.

Both cereals played and play an important traditional role in Asia, especially in India, where they became established as crops between 4000 and 3500 years ago (Boivin and Fuller 2009; Fuller 2003). However, these cereals derived from distinct centers of domestication in sub-Saharan Africa, with the first known domesticated pearl millet in the western sub-Saharan Sahelian zone and sorghum in eastern Sudan (Harlan 1971, 1992; Fuller and Hildebrand 2013). Hard evidence for the domestication processes and the earliest dispersal of these cereals remained elusive until recently (Kahlheber and Neumann 2007; Manning et al. 2011; Winchell et al. 2017).

Previously, Harlan and Stemler (1976) pinpointed the eastern Sahelian zone between Lake Chad and northwest Ethiopia as the most likely center for *S. bicolor* domestication. Haaland (1999) reiterated these findings and proposed that morphologically wild sorghum was processed in the Central Sudan by the fifth millennium BC, citing the grindstones at Kadero I, Um Direiwa, ceramic impressions as evidence. Haaland (1996) has also hypothesized that, in the Sudan, sedentism led to more intensive cultivation of sorghum as local resources became depleted, thus initiating the domestication process.

For *P. glaucum*, Tostain (1992), Fuller (2003), and Neumann (2003) in particular have hypothesized that there may have been more than one center of domestication west of Lake Chad. By the early second millennium BC, morphologically domesticated pearl millet was widely cultivated across sub-Saharan West Africa Fuller 2007; Klee et al. 2004; Neumann 2005). However, the presence of the cereal in India by ca. 1700 BC (Fuller 2003) indicated that the domestication process had started earlier and that there was a fairly rapid eastward dispersal.

Recently, Mercuri et al. (2018) have documented a long history of inferred cultivation of wild cereals in the Central Sahara in the Acacus Mountains area. Here at the site of Takarkori, Libya, they report evidence for Early Holocene gathering of wild sorghum (*S. bicolor* subsp. *verticilliflorum*) and *Pennisetum* in the Late Acacus (7500–7100 BC), alongside Panicoid millet grasses, including *Brachiaria*, *Urochloa*, *Digitaria*, and some *Panicum*. Wild sorghum use seems to have decline and disappeared by ca. 6400 BC, while later occupations provide evidence for the cultivation of other small millets, especially *Echinochloa colona* and *Panicum laetum*, is indicated by large quantities, evidence for storage and morphological change in grain shape/size, especially between 4800 and 3800 BC (Mercuri et al. 2018). In this case, it is argued that cultivation focused on species that produced smaller grain prolifically and which tolerated disturbance from grazing. Although these grains did not become the cereals of later agriculture here or elsewhere in Africa, they attest to multiple regional traditions of independent early plant cultivation in the middle Holocene of Saharan/Sahelian Africa, of which West African pearl millet and East African sorghum were other examples.

**Fig. 1:**
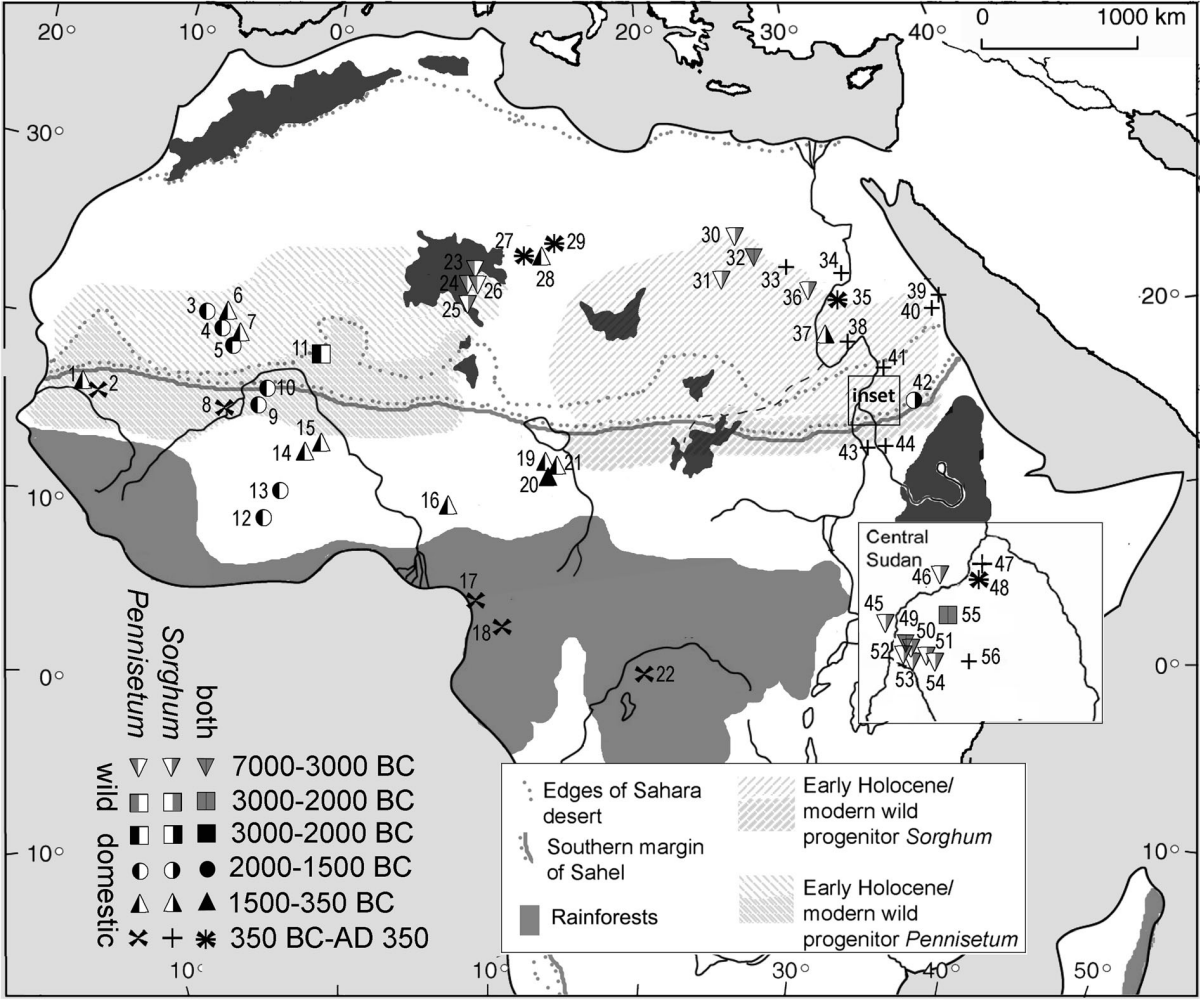
Map of the archaeological distribution of sorghum and pearl millet finds in northern Africa up to AD 350. The median age of the earliest phase of occurrence for each site is represented. Wild forms are indicated in gray, domesticated morphology indicated in black. Sites: 1. Waladé; 2. Cubalel; 3. Dhar Tichitt; 4. Dhar Oualata; 5. Djiganyai; 6. Oued Chebbi; 7. Oued Bou Khzama; 8. Dia Shoma; 9. Ounjougou (Varves West); 10. Windé Koroji; 11. Karkarichinkat; 12. Boase B5C; 13. Birimi; 14. Oursi; 15. Ti-n-Akof; 16. Nok sites; 17. Bwambe-Sommet; 18. Aband Minko’o; 19. Ganjigana; 20. Mege; 21. Kursakata; 22. Boso-Njafo; 23. Ti-n-Torha/Two Caves; 24. Uan Tabu; 25. Takarkori; 26. Uan Muhuggiag; 27. Tinda B; 28. Zinchechra; 29. Jarma; 30. Farafra Oasis; 31. Abu Ballas; 32. Dakhleh Oasis; 33. Kharga Oasis; 34. Wadi Qitna; 35. Qasr Ibrim; 36. Nabta Playa; 37. Kawa; 38. Umm Muri; 39. Berenike; 40. Shenshef; 41. Dangeil; 42. Kasala; 43. Jebel Tomat; 44. Abu Geili; 45. Shaheinab; 46. El Kadada; 47. Meroe; 48. Hamadab; 49. El Zakiab; 50. Kadero; El Zakiab 51. El Mahalab; 52. Umm Direiwa; 53. Sheikh el Amin; 54. Sheikh Mustafa; 55. Shaqadud; 56. Jebel Qeili. (map: D.Q. Fuller)

Against the backdrop of these models, the work of Winchell et al. (2017) on cereal impressions in Butana Group ceramics places the start of the sorghum domestication process in the eastern Sudan in at least the fourth millennium BC (Fig. 1), which Beldados et al. (2018) argue continued down to the start of the second millennium BC. Congruent with evidence in other cereals, such as Near Eastern wheat and barley or Chinese rice (Fuller et al. 2014), the evolution of morphologically domesticated sorghum appears to have been a protracted process. It is plausible that the process for domesticating pearl millet was broadly contemporary in the western Sahel zone, where fully domesticated varieties of pearl millet have been found in southeastern Mali as early as 2500 BC and unambiguously by 2000 BC (Manning et al. 2011; Manning and Fuller 2014). Potential areas where pearl millet may have been first domesticated may lie in the vast unexplored regions of the Sahel and southern margins of the Sahara including Mauritania, Mali, and parts further east, such as northern Niger. Nevertheless, Magid (1991) tentatively identified pearl millet perhaps as early as 2900 BC from Shaqadud Cave, a little over 50 km east of the central Nile Valley in the western Butana region of the Sudan, raising questions about when, and from where, pearl millet came to Sudan and first became a cultivar alongside sorghum.

As argued previously by Beldados (2012, p. 101) and more recently by Winchell et al. (2017), the environmental and social conditions present in the fertile savanna zone of the southern Atbai (eastern Sahel, Sudan) was optimal for the domestication of sorghum. This region had well-established early ceramic-producing cultures of hunter-foragers since the mid-sixth millennium BC (Pre-Saroba and Saroba Group, Table 1). These peoples eventually became sedentary more than 2000 years later and were associated with a ceramic-bearing culture defined as the Butana Group (Winchell 2013). Subsequently, the successor Gash Group established a socially complex administrative center at Mahal Teglinos near Kassala. They conducted trade with the states of Egypt and Kerma and other cultures of Lower and Upper Nubia, as well as with groups further east in the Eritrean-Ethiopian highlands and across the Red Sea into the southern Arabia Peninsula (Fattovich 2010, 2012; Bard and Fattovich 2013). Here, it is proposed that the Gash Group was the culture, which may have been most responsible for the transference of native African summer rainfall cereals to India by the end of the third millennium BC, as it had contact with other cultures in Arabia, especially coastal and fishing people, who, in turn, would have had contact with the Indian continent further to the east (Boivin and Fuller 2009). At about the same time, domesticated pearl millet may have been present at Shaqadud Cave to the west, but its presence or that of other cereals can only be verified by new samples from undisputed, securely dated stratigraphic contexts from fresh excavations. Nevertheless, pearl millet was present along with sorghum at Kassala, ca. 1900 BC (Beldados et al. 2018). Thus, it is the right time to reassess the evidence of the presence of these two cereals in the far eastern Sahel.

**Table 1:** A chronological summary of the southeastern Sudan. (From Manzo 2012, Winchell 2013)

Period	Southeastern Sudan
6000–5000 BC	Pre-Saroba
5000–4000 BC	Malawiya Group (Saroba Phase)
4000–3800 BC	Malawiya/Butana Transition
3800–2700 BC	Butana Group (Kassala Phase)
2700–1700 BC	Gash Group (Kassala Phase)
1700–500 BC	Jebel Mokram (Kassala Phase)
500 BC – AD 500	Hagiz Group (Jebel Taka Phase)

## Background on the Cultivation Regimes in Northeast Africa

Egypt and north and central Nubia relied on winter cultivation of wheat and barley (Madella et al. 2014; Out et al. 2016). Such systems would have become increasingly difficult as one moved south (where winters were warmer) and away from the Nile (where rains were scarce or focused in summer). Beyond crops, livestock were fundamental to the economy, including goats and sheep as well as cattle (Barich 2016; Brass 2018; Grigson, 1991, 2000; Linseele et al. 2014; Shirai 2013; Stock and Gifford-Gonzalez 2013). By contrast, the Sahel and northern savannas, from Mauritania to Sudan, were the primary habitat since at least the end of the Pleistocene for a host of native African summer rainfall wild cereals including sorghum and a variety of millet species (*Pennisetum*, *Digitaria*, *Panicum*, *Brachiaria*, etc.) (Clark 1980; Harlan 1971; Marshall and Hildebrand 2002; Neumann 2005).

Across the southern part of the Sahara, during the wetter early Holocene, hunter-gatherer groups gathered a wide suite of native savanna grasses, or wild millets, including *Brachiaria* spp., *Digitaria* spp., *Echinochloa* spp., *Setaria* spp., *Sorghum* spp., *Urochloa* spp., and more occasionally *Pennisetum* (Barakat and Fahmy 1999; Barich et al. 2014; Mercuri 2001, 2008; Mercuri et al. 2018; Stemler 1990a, b; Wasylikowa 1992; Wasylikowa and Dahlberg 1999). It was among such communities that ceramics appeared as a food processing technology, by ca. 8000 BC (Close 1995; Garcea 1998, 2005; Huysecom et al. 2009; Jesse 2003). This process is particularly well-documented from several sites in the Acacus region of southern Libya that have produced statistically robust plant assemblages documenting savanna grass foraging (Castelletti et al. 1999; Mercuri 2001; Mercuri et al. 2018; Olmi et al. 2011; Wasylikowa 1992).

**Fig. 2:**
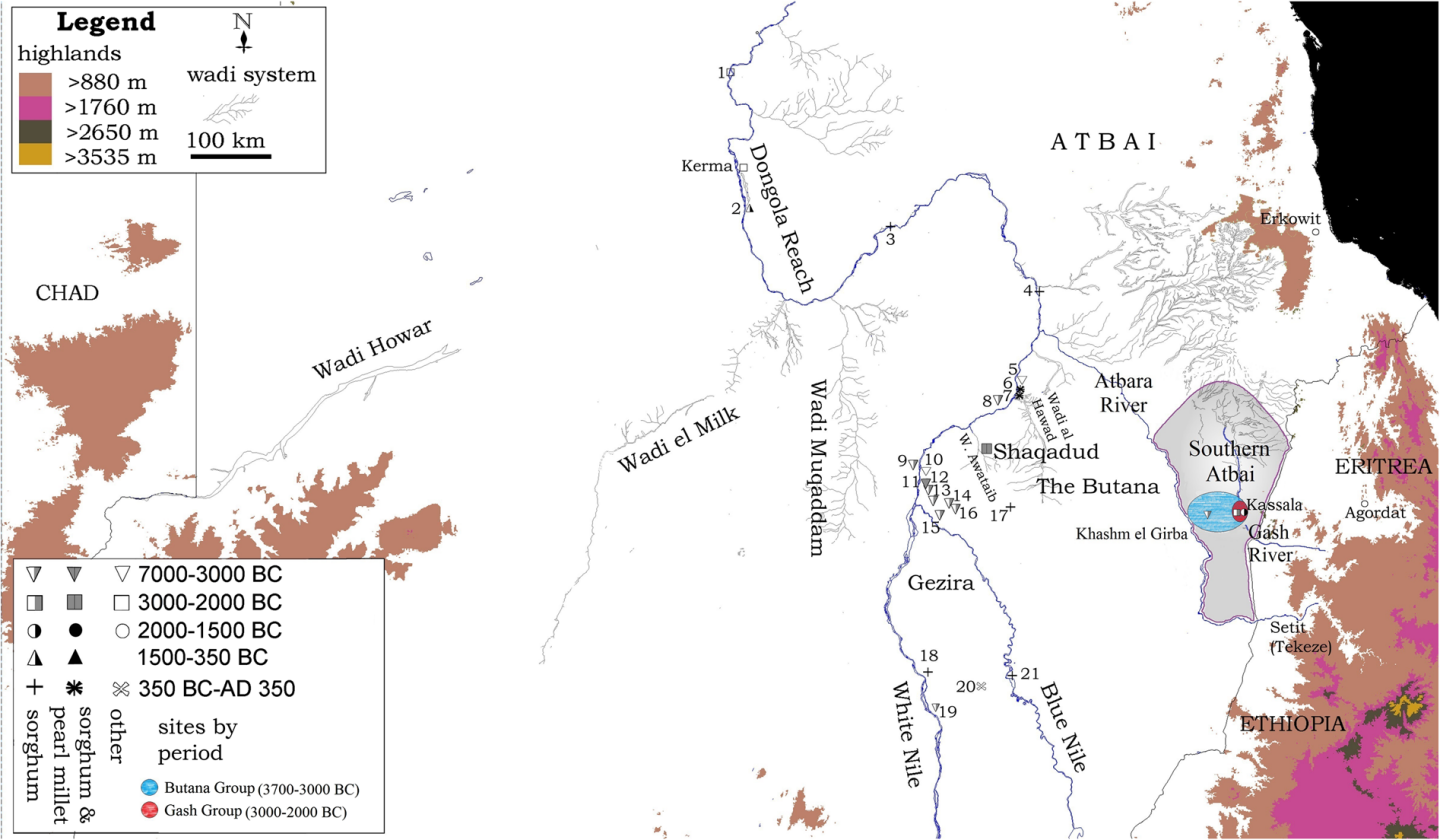
Map of the Sudan, relating regions and sites discussed. The presence of sorghum in sites of various period is indicated. Sites: 1. Sai Island; 2. Kawa; 3. Umm Muri; 4. Dangeil; 5. Es Sour; 6. Meroe; 7. Hamadab; 8. El Kadada; 9. Sheheinab; 10. Geili; 11. El Zakiab; 12. Kadero; 13. Umm Direiwa; 14. El Mahalab; 15. Sheikh Mustafa; 16. Sheikh el Amin; 17. Jebel Qeli; 18. Jebel Tomat; 19. Rabak; 20. Jebel Moya; 21. Abu Geili

Among the many smaller-grain wild millets, larger-grained cereals like wild sorghum may have sometimes been favored. As already noted, this was not the case in the Acacus region, where small-grained species, with “weedy” characteristics such as tolerance for grazing, were favored by the mid-Holocene (Mercuri et al. 2018). In contrast, however, larger-grained sorghum was favored sometimes, including in the earlier Holocene, and pre-pastoral, Late Acacus period (ca. 6000 BC), where it is known in large seed accumulations (Mercuri et al. 2018). Wild sorghum also appears to have been a favorite in the Western Desert of Egypt alongside several smaller millet grasses, at sites such as Nabta Playa E-75-6 (8000–7000 BC), Farafra Oasis (ca. 5900 BC), Dakhleh Oasis (ca. 6400–5600 BC), and Abu Ballas (ca. 4000 BC) (Barakat and Fahmy 1999; Thanheiser 2011; Wasylikowa and Dahlberg 1999). While further south, early ceramic-bearing cultures associated with the Early Khartoum (Khartoum Mesolithic) may have utilized a similar breadth of naturally occurring summer rainfall cereals (Panicoids and sorghum), including upstream from the confluence of the Nile and Atbara Rivers and further beyond the 6th Cataract (Fernández et al. 2003; Haaland 1996), although this has yet to be found at contemporary Al Khiday sites south of Khartoum. In the following millennium, Early Neolithic peoples in the Central Sudan region north of modern-day Khartoum left evidence in ceramic impressions for wild millet and wild sorghum use. This is best documented along the Nile just north of Khartoum and the lowermost Blue Nile (Fig. 2). These finds of sorghum impressions range from as early as ca. 5700 BC at Kadero I to perhaps at late as ca. 3750 BC at Umm Direiwa I (Haaland 1981; Magid 1982, pp. 97–98; Abdel-Magid 1989, Magid 1991, pp.194–195; Sadig 2010, pp. 72–74; Stemler 1990a, b). Stemler (1990a, b) reported a single possibly domesticated type spikelet of sorghum from Umm Direiwa. Recently two new assemblages of plant impressions in pottery have been studied (Beldados et al. 2018; Winchell et al. 2017), both of substantially larger sample size than previous datasets, which show that the domestication process for sorghum had begun by the fourth millennium BC and was still underway by the start of the second millennium BC.

## Overview: Domestication of Pearl Millet and Sorghum

Domestication in cereals is a set of genetic and morphological adaptations that make the domesticated crop more suitable to cultivation, including harvesting, storage, and planting, than its wild relatives (Fuller 2007; Harlan 1992; Harris and Fuller 2014; Abdel-Magid 1989). A key change is the loss of wild seed dispersal by shattering of the panicle or ear, which in the wild is what disperses seeds. Instead, domesticated cereals retain their grain and require threshing. Other changes associated with domestication include loss of dormancy and increase in grain size. In the study of wheat(s) and barley domestication across southwest Asia, the gradual evolution of non-shattering and grain size are well-documented as taking place over 3000–4000 years, starting in the tenth millennium BC (Fuller et al. 2014). Similarly, the evolution of non-shattering in Chinese rice has been documented as a protracted process taking some millennia (Fuller et al. 2009). In contrast, clear archaeological evidence for the same changes in African cereals has been lacking, i.e., there are no chronological sequences that document these gradual changes. Nevertheless, in later periods such as the Meroitic (late first millennium BC), evidence for morphologically domesticated sorghum is clear (Fuller 2014; Rowley-Conwy 1991). The lack of earlier domesticated evidence led to speculation that sorghum was a late domestication, perhaps only in the first millennium BC (Rowley-Conwy et al. 1997; Wigboldus 1996). Abdel-Magid (1989) and Haaland (1995, 1999) argued that a late domestication could be due to sorghum being cross-pollinated in its natural environment. More recently; however, Allaby et al. (2010) and Fuller (2014; Fuller and Boivin 2009) have challenged that, as with other cereals around the world, intensive cultivation would still lead to the evolution of morphological domestication traits, regardless of self-pollination (e.g., wheat and barley) or cross-pollination (e.g., rice).

Wild millets are clump-forming plants. They lend themselves to management and early cultivation by mobile groups of hunter-foragers or pastoralists (Fuller et al. 2016). While they retain wild-type dormancy they are easy to store and suit seasonal mobility (Mercuri et al. 2018). Cultivation would then represent a strategy to create new clumps or expand existing patches, a pattern argued to be relevant to not only millet domestication in north China (Stevens and Fuller 2017) and southern India (Murphy and Fuller 2016) but also in agreement with the evidence for small millet cultivation in the central Sahara (Mercuri et al. 2018). Thus, cultivation of millets in the central Sahara probably preceded sedentism. This appears also to have been the case with western Saharan pearl millet (*P. glaucum*) too. Semi-sedentary sites appear to have developed in the Tilemsi Valley of northern Mali (2500–2000 BC) after pearl millet was already domesticated, or at least well on its way (Manning and Fuller 2014). A key question is whether a similar pattern holds for sorghum, as well the extent to which mobile pastoralists were responsible for early sorghum cultivation and domestication.

## Pearl Millet Domestication and Dispersal to India

Research in the Lower Tilemsi Valley in eastern Mali (western Sahel) has pushed back the date for early domestication of pearl millet to 2500–2000 BC (Manning et al. 2011; Manning and Fuller 2014). In the first of two phases of early food production, starting ca. 2500 BC, the Valley was used seasonally by pastoralists coming from elsewhere (perhaps from the north) for seasonal fishing, represented by sites such as Karkarichinkat North. In these sites, millet chaff-tempered ceramics were rare, and no definitive evidence for domesticated-type chaff was found. However, two grains of pearl millet resembled the domesticated shape and one was directly-dated by AMS to ca. 2500 BC, suggesting that the mobile groups had access to millets probably cultivated elsewhere in their territories (Manning and Fuller 2014, p. 79). By 2000 BC, the populations were semi-sedentary and chaff-tempered ceramics comprised up to 30% of the assemblages; domestication traits such as non-shattering grains were widespread.

If the domestication rate for non-shattering in pearl millet was similar to cereals like wheat, barley or rice (Fuller et al. 2014), then fixation of non-shattering at ca. 2000 BC would imply initial cultivation around 4000 BC somewhere in the southwestern Sahara (Manning and Fuller 2014). Further to the west in eastern Mauritania, chaff-tempered ceramics indicate the presence of domesticated pearl millet by 1700 BC (Amblard and Pernès 1989; Fuller et al. 2007). The cultural traditions in this area appear to derive from their own cultural tradition, unconnected to that from the Tilemsi Valley, and thus could represent the end of a distinct trajectory of pearl millet domestication (MacDonald et al. 2009). The southward dispersal of early pearl millet then might be seen as part of the retreat of the “green Sahara” of the middle Holocene, as climate dried and pastoral populations across the Sahara decline (Manning and Timpson 2014).

To the east, African domesticated pearl millet is present in the northwestern part of subcontinent of India, below the Indus Valley in the Saurashtra peninsula (Gujarat) by at least 1700 BC, and probably earlier, perhaps 1900 BC (Boivin and Fuller 2009, p. 146; Fuller 2003, pp. 247–250; Manning et al. 2011, p. 318). The early pearl millet recovered from sites in India are of the small-grained variety, suggesting derivation from early Sahelian forms, while large-grained forms evolved independently in India (Fuller 2007). Nevertheless, based on a point of origin for domesticated pearl millet occurring in the western Sahel, the dissemination of this cultigen eastwards must have been rapid, occurring parallel to, or even before, millet cultivators settled in the Lower Tilemsi Valley.

Manning et al. (2011, p. 318) argue that such an eastward path of dissemination would have perhaps occurred along the northern Sahelian corridor through the regions of Niger, Chad, and finally the Sudan. Limited evidence in support of this has recently come to light at the Kassala site of Mahal Teglinos (ca. 1850 BC median age). Among predominant impressions of sorghum spikelets (see below) there, two examples of *Pennisetum* spikelets/involucres have been found, one each of wild and domesticated forms (Beldados et al. 2018). Boivin and Fuller (2009) have highlighted the maritime trade across the Red and Arabian Seas. It was a two-way process. Broomcorn millet (*Panicum miliaceum*), of ultimately Chinese origin, arrived in Yemen by 2000 BC and in Classic Kerma period Ukma, Nubia, by ca. 1700 BC. Broomcorn millet is absent from Mesopotamia and Egypt in this era, indicating its arrival across the Arabia Sea and/or Arabian Peninsula (Boivin and Fuller 2009; Stevens et al. 2016).

This route has been proposed for other crops of African origin as well, including sorghum and the hyacinth bean (*Lablab purpureus*), arriving in India (Boivin and Fuller 2009). The hyacinth bean is particularly widespread in peninsular India in the second millennium BC, including seven direct AMS dates (Fuller and Harvey 2006), with its geographical origins narrowed down to the Ethiopian and eastern Sudanic regions (Fuller and Hildebrand 2013; Maass et al. 2005), suggesting arrival from Africa around the same time as sorghum and pearl millet.

## Sorghum: an Eastern Sahelian Domesticate

In the far eastern Sahel lies the southern Atbai. It is bordered by the Eritrean-Ethiopian highlands to the east and Atbara River to the west. The eastern portion of the southern Atbai includes the Gash River as it drains from the Eritrean-Ethiopian highlands and ends at its inland delta, known as the Gash Delta, north of the present-day city of Kassala. Ceramic-bearing cultures have inhabited the southern Atbai since the sixth millennium BC in a long developmental sequence through the first half of the first millennium AD (Fattovich et al. 1984; Marks et al. 1986; Marks and Fattovich 1989; Manzo 2017; Mbutu 1991; Sadr 1988, 1991; Winchell 2013). Populations associated with these early ceramic-bearing cultures were fairly scattered and small, ranging as hunter-gatherers, exploiting riverine resources and hunting in the interior steppe (Fattovich and Piperno 1992; Manzo 2017, p. 19; Marks 1987; Mbutu 1991, pp. 103–104; Winchell 2013, pp. 10–12).

Subsequent cultures in the southern Atbai and their associated ceramics have been classified within a single tradition known as the Atbai Ceramic Tradition (ACT) (Fattovich 2010, p. 150; Fattovich et al. 1984; Marks et al. 1986; Marks and Fattovich 1989; Sadr 1991; Winchell 2013, pp. 12–17). The ACT has been subdivided into five ceramic groups (Malawiya, Butana, Gash, Jebel Mokram, and Hagiz), organized within three ceramic phases (Saroba, Kassala, Jebel Taka) (Fattovich et al. 1984; Marks and Sadr 1988; Winchell 2013, pp. 12–17). The earliest ceramic group associated with this tradition is called the Malawiya Group (mid-fifth millennium BC). The Malawiya Group is represented by small sites averaging 5000 m^2^ of mobile hunter-foragers who were more oriented to the interior steppe region within the southern Atbai. Ceramics of the Malawiya Group are generally similar to the widespread Khartoum Variant ceramic designs found further west in the central Nile Valley and adjacent parts in the Sahel, including various rocker-stamped and punctate designs produced by combed implements (Hays 1971; Winchell 2013, p. 110).

**Fig. 3:**
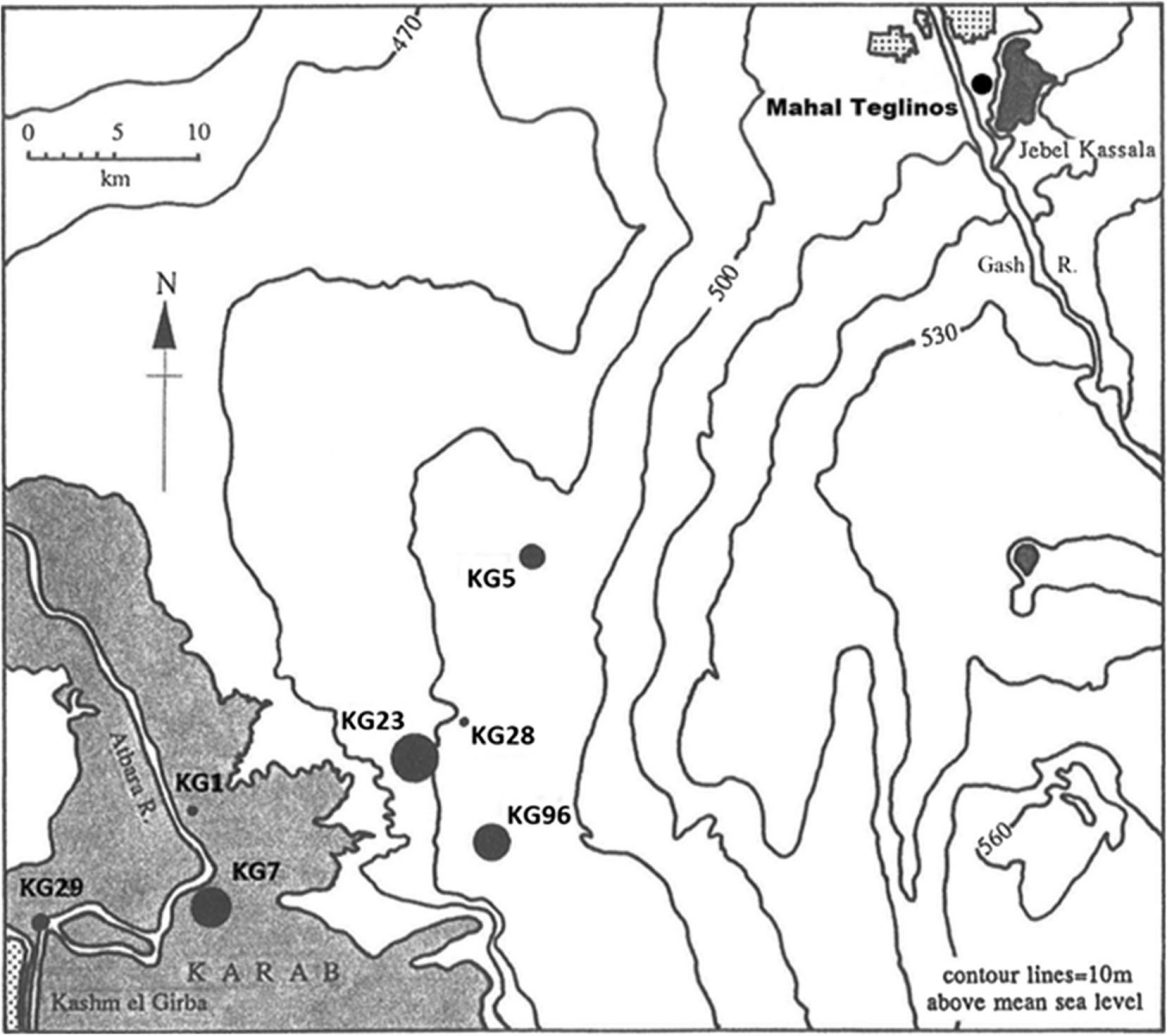
Butana Group sites across the southern Atbai (from Winchell 2013, Fig. 1.4)

At the end of the fifth millennium BC, the Malawiya Group transitions into the Butana Group. The large site-size (15,000 m^2^) of the only known transitional occupation may indicate some trend towards a more sustained settlement. From ca. 3800 BC, people associated with the Butana Group resided in significantly larger village sites, five of which range from 6 to 12 ha in size and contain midden deposits averaging 2 m in depth (Fig. 3) (Fattovich, et al. 1984, p. 180; Mbutu 1991, p. 374; Winchell 2013, pp. 13–14). Based on the massive size of the larger sites, the populations appear to have been sedentary. There may have been some ranking of individuals within the Butana Group due to the presence of prestige items, such as mace heads made from imported porphyry resembling similar artifacts associated with A-Group and Egyptian Predynastic societies (Fattovich 2010, pp. 154, 167; Mbutu 1991, p. 426). Unfortunately, the funerary evidence recovered at this time is still limited and cannot illuminate more on the social ranking of Butana Group individuals (Manzo 2017, p. 27).

The Butana Group peoples predominately hunted wild fauna and exploited large land snails and other freshwater resources (Manzo 2012, p. 8, 2017, p. 25; Peters 1986). Only later did they augment their diet with limited numbers of domesticated cattle and ovicaprids (Peters 1986), perhaps introduced from the Nile Valley (Haaland 1981, 1986, 1987), other parts of the Sahel and Sahara, or from the Red Sea Hills. Some of the authors have recently argued that the origin of the sorghum domestication process is traceable to this period, when the steppe environment was wetter (Winchell et al. 2017).

At around 3000 BC, there was a shift in Butana Group settlements. At this time, Winchell (2013, pp. 15, 102, 137–142, 146) believes that some of the Butana Group people may have also migrated to the west across the Butana grasslands and settled at Shaqadud Cave. Nevertheless, the majority settled around the center of Mahal Teglinos. The ceramics and associated culture at Mahal Teglinos are defined as the Gash Group which occupied the center from ca. 2700 to 1700 BC (Winchell 2013, pp. 15, 102, 137–142, 145–146; Manzo 2012, 2017, pp. 33–43).

**Fig. 4:**
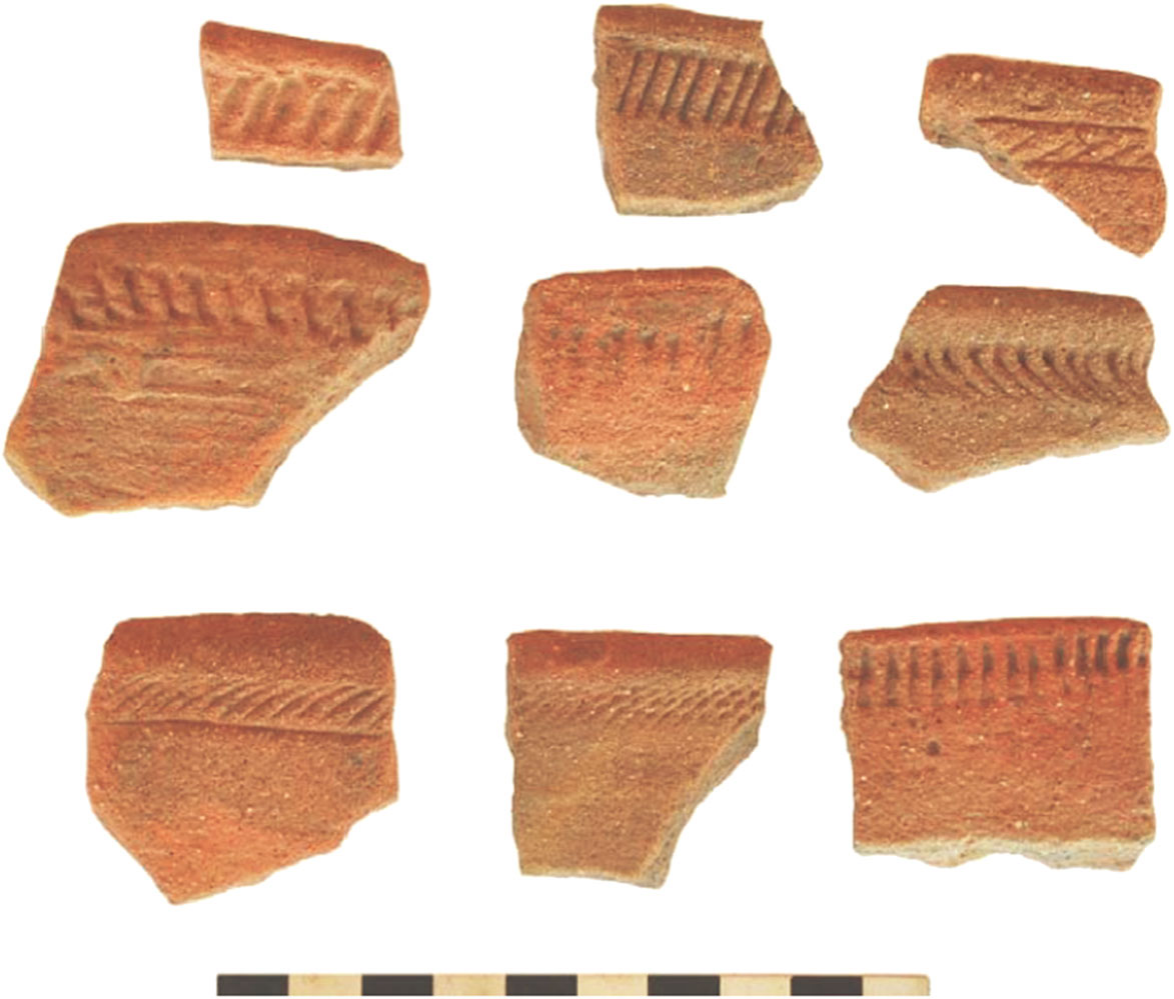
Rim sherds of Classic Gash rim banded bowls and cups (from Manzo 2012, Fig. 83)

Sadr (1991) modeled the Gash Group as having practiced a mixed farming and herding economy, although detailed subsistence data are limited. The inland Gash Delta at this time would have also been more favorable than the interior steppe environment for the continued cultivation of sorghum which had begun with the Butana Group. Some of the authors have also argued that domesticated sorghum was present during the Gash and successor Jebel Mokram groups (Beldados et al. 2018; Beldados and Constantini 2011). The social structure of the Gash Group demonstrated some inklings of an incipient state-like system of organization, including: (1) a sedentary center at Mahal Teglinos (near the modern-day city of Kassala) and associated nucleated settlements, cemeteries, and associated stelae monuments; (2) administrative stamping devices (mushroom-shaped clay stamp seals); (3) standardized pottery (Fig. 4); and (4) architecture consisting of a mud-brick building with storerooms (Fattovich 2010, pp. 154–155, 161–162; Manzo 2017, pp. 36–43). Egyptian jars dating to this period have also been found at Mahal Teglinos (Fattovich 2010, p. 161; Manzo 2017, p. 35). Overall, the inhabitants at Mahal Teglinos were trading with Egypt, Nubia, and the southern Arabian Peninsula during the Early Gash Group phase (ca. 2800–2500 BC), then with Kerma during the Middle (ca. 2500–2100 BC) and Classic (ca. 2100–1900 BC) Gash Group phases, and again with Egypt, Nubia, and the southern Arabia Peninsula in the late Gash Group phase (ca. 1900–1700 BC) (Fattovich et al. 1988–1989; Fattovich 1989a,b,c, 1991a, b, 1993a, b, c, 1995, 2006, 2007, 2010; Manzo 1997, 2010, 2012, 2017, pp. 33–35; Sadr 1991).

**Fig. 5:**
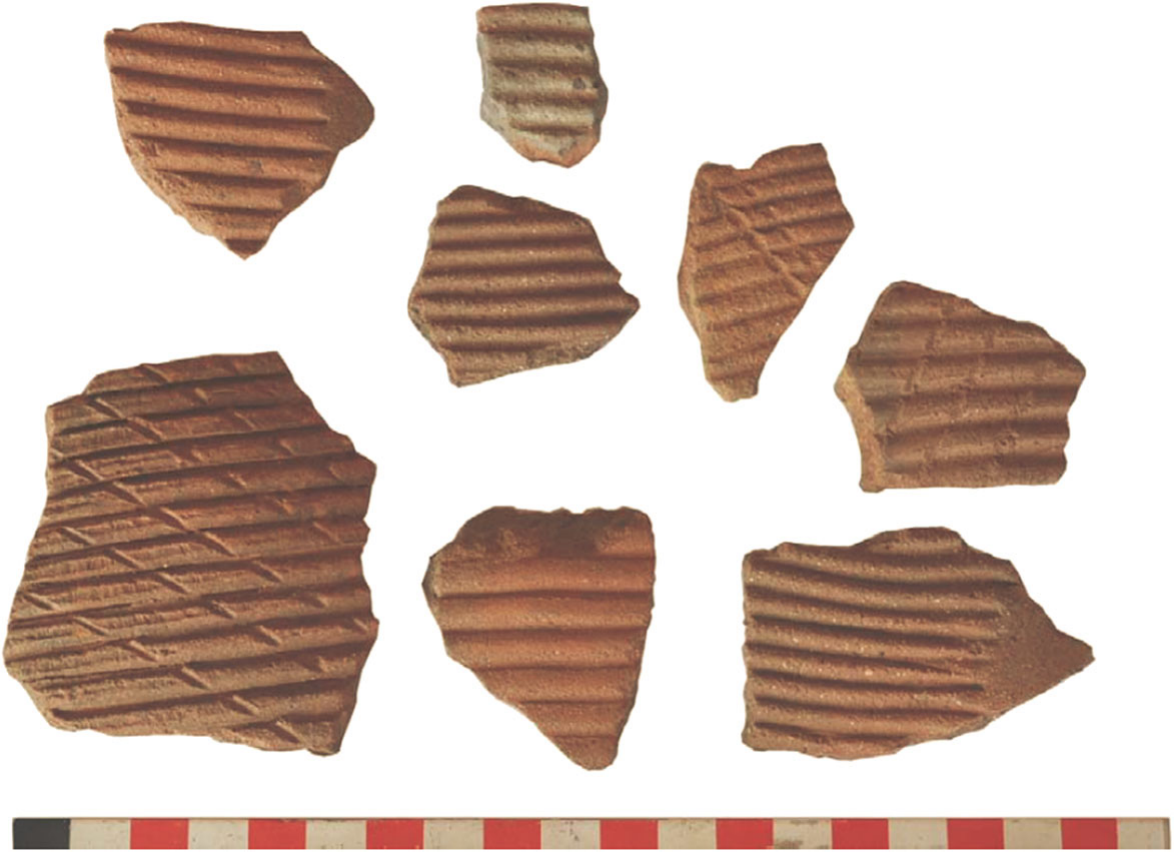
Horizontally combed and grooved Jebel Mokram Group sherds (from Manzo 2012, Fig. 85)

**Fig. 6:**
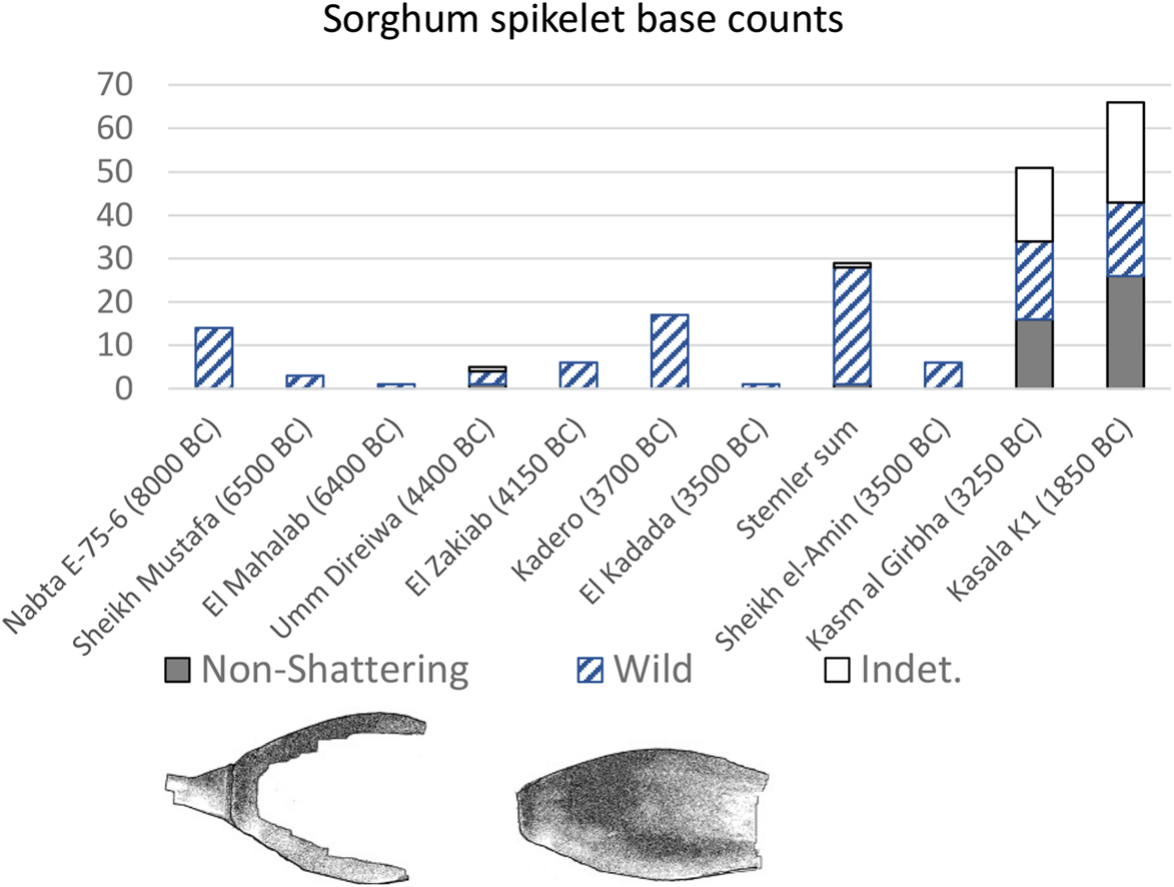
Quantitative summary of archaeological spikelet base data for early sorghum from eastern Saharan/Sahel regions, based on macro-remains from Nabta Playa (Wasylikowa and Dahlberg 1999) and impressions from Sudanese sites (Stemler 1990a, b; Abdel-Magid 2003; Winchell et al. 2017; Beldados et al. 2018). These sites are selected based on the presence of quantitative data on spikelet base morphology

As with both the Butana and Gash Groups, the Jebel Mokram Group (Fig. 5) contain seed and chaff tempered ceramics including impressions of sorghum spikelets (Beldados and Constantini 2011, Beldados 2012, pp. 100–101). While many of the spikelets resemble wild, freely shattering morphotypes of *S. bicolor* subsp. *verticilliflorum*, some also represent domesticated morphotypes, with non-shattering threshed spikelets and husks. Recent study of Late Gash impressions in burned clays, perhaps from storage vessels, from lower layers at Mahal Teglinos K1 indicate a mix of wild and domesticated morphotypes dating to 1960–1760 BC (Beldados et al. 2018). This assemblage of impressions appears slightly more domesticated, i.e., further along the trajectory in the Sudan, than at earlier KG23 (Fig. 6). In addition, unlike the earlier KG23 assemblage which included the domesticated type with torn rachillae, which can occur in very low frequencies in wild populations or with immature harvests, Mahal Teglinos included also “rip scars,” a spikelet-based morphotype found only in domesticated populations. This also testifies to the Late Gash K1 sorghum population being further along the evolutionary path to domestication than the Butana Group sorghum of KG23 (Beldados et al. 2018). Two examples, one domesticated and one wild type, also indicate the presence of pearl millet (*P. glaucum*) in this late Gash Group burned clay.

## Indications of Evidence for Cultivation Activity in the Butana Group

The relatively sudden increase in site size among Butana Group settlements, beginning sometime after 3800 BC, points to a departure from hunting and gathering that had been the mainstay within the region for at least several millennia. With radiocarbon dates and the seriation of ceramics within the midden deposits, two of the largest Butana Group sites may have been occupied during the same time during the earlier phase (ca. 3800–2900 BC), while three of the remaining sites may have been more heavily occupied during the later phase (ca. 3000–2600 BC) (see Fig. 4.1 in Sadr 1988, p. 94; Sadr 1991; Winchell 2013).

**Fig. 7:**
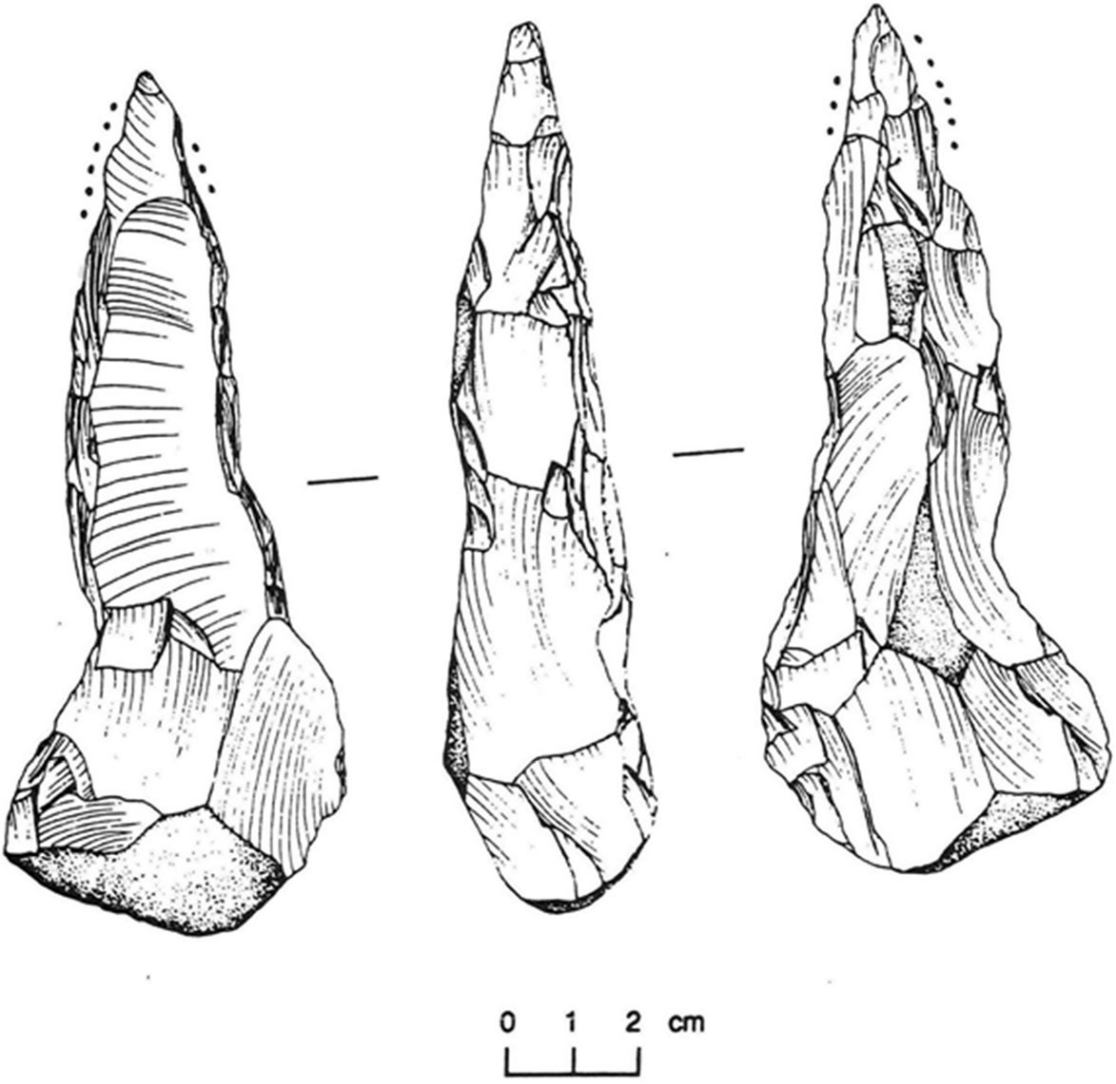
Chipped stone pick of the Butana Group (from Mbutu 1991, Fig. 2.12; reproduced with permission)

The Butana Group sites contain a rather unique type of artifact known as picks (Fig. 7). These accounted for about 15% of all the lithic tool-types identified. They were manufactured from hand-sized cobbles of chert collected from Atbara River gravels, and fashioned with a pointed tip on one end and left un-flaked on the butt (Mbutu 1991, pp. 277–278). The picks are long and narrow (mean length 62 mm, mean width 26 cm) with tips that are trihedral in cross section. Of the 110 picks recovered from ten Butana Group sites, only 15 were complete. Most of the complete picks were heavily used. They had battered tips, with ground or re-sharpened (Mbutu 1991, p. 278). Winchell’s former PhD colleague from Southern Methodist University, the late Steve Mbutu, argued in his doctoral thesis on the Butana Group stone tools that the use of these picks would have been for breaking up the soil for cultivation (Mbutu 1991, pp. 374–376, 409; Sadr 1991; Shiner 1971).

Among the other ground and polished stone implements in the Butana Group assemblages are stone rings that may have been used as weights for digging sticks, especially in softer, sandier soils. Also, lunates, accounting for 10% of the recovered attributable lithics, were proposed by Mbutu (1991, pp. 166–67, 372–73) as an aid in the cultivation of cereals, used as hafted sickle blades, although they could also have been manufactured as arrow tips for hunting.

Overall, determining the presence of sickle gloss on the geometrics proved to be non-conclusive due to several factors involving raw material used (most were made from agate which has a shiny surface) and post-depositional sand-polishing (Mbutu 1991, p. 407). However, a backed curved blade recovered from the lower midden deposit (at a depth of 150 cm, dated to near the start of the Butana Group) from the largest site (KG23) did have evidence for sickle gloss (Mbutu 1991, p. 407).

A host of grinding implements (made from granite, sandstone, quartz, and quartzite) were also recovered from Butana Group sites, ranging from large querns, to smaller grinding basins, to handstones (Mbutu 1991, pp. 175–177). Although the use of grinding stone does not confirm whether groups were actually engaged in plant cultivation, their presence supports the fact that Butana Group peoples were grinding and processing plant resources, probably grains, for food (Mbutu 1991, p. 409), and along with the presence of heavy grinding stones, remnants of daub from structures, and bulky, thick-walled ceramic vessels, point to a more sedentary existence (Mbutu 1991, pp. 376–377; Winchell 2013, pp. 159–163).

**Fig. 8:**
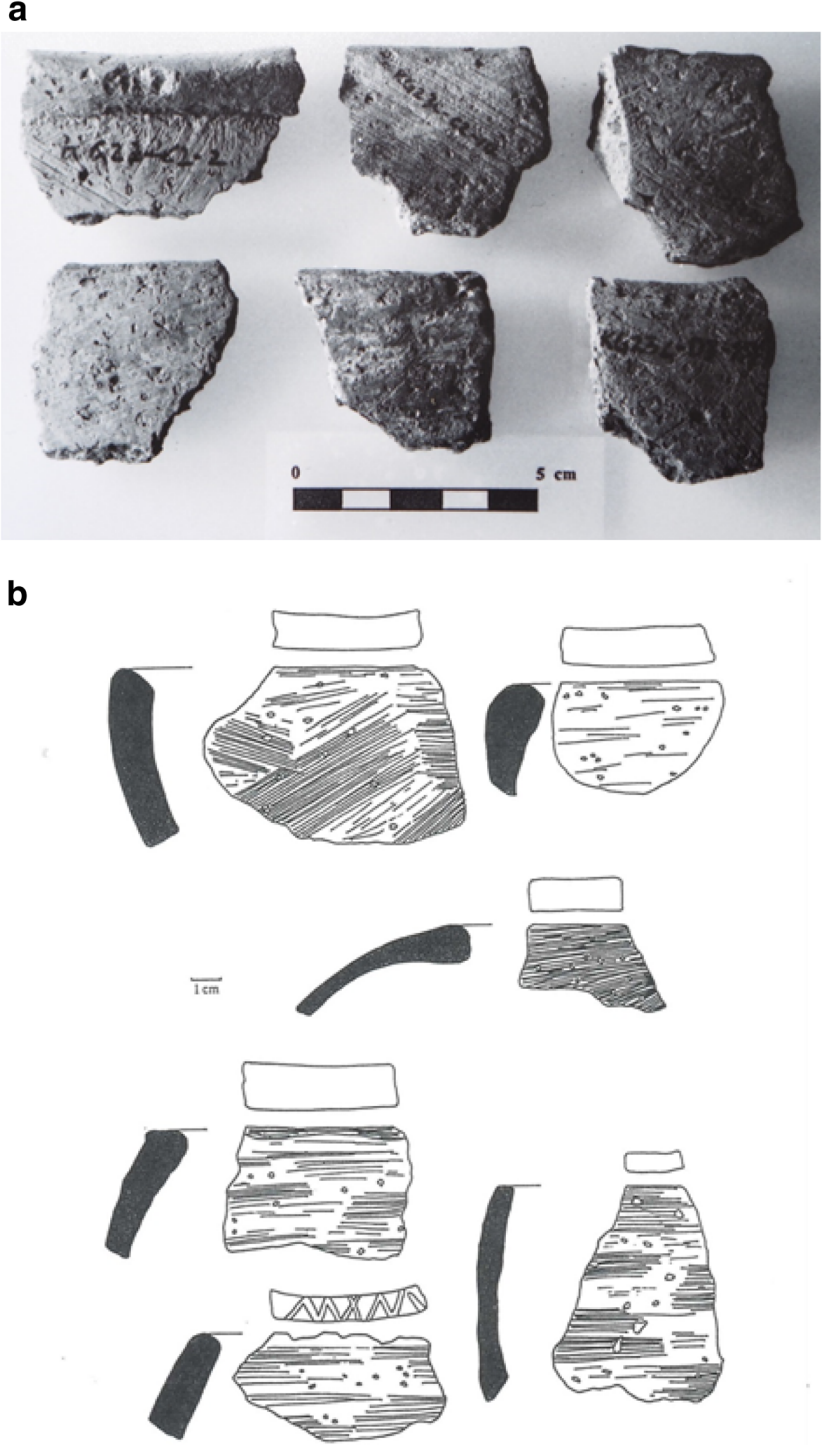
**a** and **b** Seed-chaff tempered ceramics (Khordhag Plain type) of the Butana Group. ([b] from Winchell 2013, Fig. A.37)

Despite the lines of circumstantial evidence for cultivation in terms of plausible tillage and harvesting tools, definitive evidence for cultivation comes from archaeobotanical signs of crops undergoing morphological evolution towards a domesticated form (Harlan et al. 1973; Fuller 2007). Fully domesticated cereals are essentially dependent on humans for seed dispersal, as the appearance of such morphotypes in high frequencies implies an ecology of human harvesting and sowing that makes such morphotypes adaptive. Chaff-tempered ceramics, therefore, have the potential to preserve evidence not only for crop-processing by-products being used in pottery production but also for evolving cereal morphology. At Butana Group sites are associated seed and chaff tempered ceramics referred to as the *Khordhag Plain* type (Fig. 8a–b). The *Khordhag Plain* type consists of ca. 10% of the sherds recovered from throughout the midden deposits of the Butana Group sites (Winchell 2013, pp. 36–70). The pastes associated with this type contain relatively few mineral inclusions but were tempered predominately with large amounts of seeds (or spikelets, grains in the husk), along with some free chaff. The *Khordhag Plain* type is present within all of the large Butana Group middens and appear in proportionately larger numbers during the later phases.

A total of 91 body sherds of the *Khordhag Plain* type from the largest Butana Group site, KG23 were examined by Winchell et al. (2017). Of these, 65 sherds produced 279 identifiable plant impressions of which 249 were *S. bicolor*. Both wild and domestic types of sorghum were found among the sorghum impressions and with the total range of impressions including panicles, grains, spikelets, rachillae, and associated chaff indicate that populations associated with the Butana Group at KG23 (3500–3000 BC) were in the pre-domestication stage of the domestication process as defined by Harris and Fuller (2014). The proportions of domestic to wild forms of sorghum recovered from the *Khordhag Plain* sherds are roughly equal (Fig. 6). Based on the overall plant impressions from these sherds, the presence of non-shattering domesticated and shattering wild spikelets, along with immature spikelets, would indicate that Butana Group populations were harvesting mixed stands of wild and domesticated morphotypes of sorghum while some of these panicles were still green. The green harvesting technique would have been used to acquire as much grain as possible, especially when the stands of sorghum were mixed with both domestic and wild varieties; this is similar to the evidence found for rice when it was still partly shattering and undergoing domestication (Fuller et al. 2009). They were also threshing the harvest to separate both the domestic and immature spikelets from the panicle, indicated by the presence of impressions of fine straw/leaves used for tempering the vessels. Subsequent dehusking is indicated by broken husks from which the grain had been freed (Winchell et al. 2017). By-products of both threshing and dehusking would have been separated by winnowing, and it is these winnowing by-products that were utilized by some potters in producing Khordhag Plain vessels.

## A Brief Survey of the Rest of the Sudan

There were many contemporary cultural groups occupying the Sudan in this Sahelian ecological zone that could have domesticated native African crops there, or incorporated cultivars coming from the western Sahel, especially during the third through second millennium BC (see, for example, Edwards 2004; Fernández et al. 2003; Garcea and Hildebrand 2009; Honegger 2003, 2004a, b, c, d; Jesse 2010; Manzo 2012, 2017; Sadig 2010; Salvatori and Usai 2008). Regions of particular interest in the Sudan involving a potential Sahelian route for the dissemination of cultigens and where there is the presence of well-documented prehistoric occupations dating between the beginning of the second and mid-third millennium BC (or earlier) would include the Wadi Howar, Dongola Reach of the Nile Valley, central Nile Valley, Butana (including Shaqadud), and southern Atbai including the Gash Delta. The archaeology of each of these areas is reviewed briefly below with an aim to track, in particular, the early use of sorghum or sorghum together with pearl millet.

### Wadi Howar

The Wadi Howar extends from the south Libyan Desert in western Chad to the Nile Valley, about 300 km south of the 3rd Cataract. Remains of ceramic-bearing cultures date from ca. 5200 to 1100 BC (Jesse 2004). These cultures have been divided into three ceramic horizons: Dotted Wavy Line and Laqiya Horizon (ca. 5200–4000 BC); Leiterband Horizon (ca. 4000–2200 BC); and Handessi Horizon (ca. 2200–1100 BC). During this latter period, Wadi Howar was finally abandoned due to increased dying conditions (Neumann 2005, p. 260). Although wood charcoal evidence has documented the environments in which these groups lived (Neumann 1989a, b), neither seed finds nor impressions in the ceramics have provided evidence for plant exploitation. In parallel with other parts of the Sahara-Sahel region, we can expect gathering of wild savanna grains to have been part of the economy. It is possible that cultivation could have been adopted from adjacent areas in either the later Leiterband or Handessi horizons, but the unstable and drying environment would not have allowed for such traditions to persist over the long-term.

### Dongola Reach

The northern Dongola Reach area along the Nile River is approximately 250 km north of where Wadi Howar entered the Nile. Groups associated with the Pre-Kerma culture (ca. 3500–2500 BC) were keeping domestic cattle and cultivating and storing domesticated emmer wheat and barley (winter rainfall crops) in the Nile Valley, which had been disseminated upriver from Egypt. These cereals, together with some wild seeds (including wild *Echinochloa*), were recovered on Sai Island (between the 2nd and 3rd Cataracts) in intact storage pits, suggesting seasonal cultivation (Garcea and Hildebrand 2009, p. 319; Geus 2003, 2004; Hildebrand 2007). Similar pits, probably also for storage, characterize the later Neolithic, which has Pre-Kerma characteristics as far upstream as the 4th Cataract, as indicated by finds on Umm Melyekta Island (Fuller 2004a). Unfortunately, none of the storage pits on Melyekta was found intact with its grain contents.

The processing of winter rainfall cereals, such as wheat (*Triticum dicoccon*) and barley (*Hordeum vulgare*) for bread foods at Kerma, is also well attested by cereal remains located in tombs, batteries of ovens associated with bakeries, and large numbers of bread molds found throughout the compound (Bonnet 1988, 2014; Chaix and Grant 1993, pp. 402–403). Interestingly, there is no evidence for the cultivation of African native, domesticated, summer rainfall cereals anywhere in the northern Dongola Reach area, including the center at Kerma during this time (Thompson et al. 2008, p. 379). Systematic archaeobotanical evidence from Kerma is lacking, but there is some recent evidence for barley (Cappers 2015). More than two millennia earlier, the phtyolith evidence from dental calculus of skeletons in Cemetery R12 indicates some processing and consumption of wheat and barley by ca. 5000 BC (Madella et al. 2014; Out et al. 2016). Taken together these data point to the key role for alluvium-cultivated winter cereals in the Neolithic to Bronze Age Dongola Reach.

For the planting of summer rainfall crops, the Nile in most places would have been too low during the spring and summer months. Only after irrigation methods were later introduced (including the use of the *shaduf* and later the *saqia* for extracting waters from lower elevations) in the Napatan to early Post-Meroitic (750 BC–AD 400) did cultivation of summer rainfall cereals become more feasible (Edwards 1996; Fuller 2004b, 2015).

Van Ziest (1987) has argued for the presence of small amounts of broomcorn millet (*P. miliaceum*) first domesticated in China, at the Kerma-period site at Ukma. Its presence later at Kawa (first millennium BC, Napatan Period), indicates that it remained part of the cultivar diversity in this region (Fuller 2004b). Broomcorn millet has a very short growing season (about 3 months) and was likely to have been a minor cultivar of the spring/summer, as a catch crop to provide some extra returns during the lowest period of water in the Nile.

### Central Nile Valley (Late Neolithic)

The Central Sudan, including the Shendi Reach, is another possible candidate area to seek evidence of native African cultigens. The Late Neolithic settlements of Kadada, Geili, Es Sour, and others were occupied by populations engaged in herding of cattle, sheep, and goats and were interacting with groups further down the Nile River who were herders and agriculturalists in Lower Nubia and Predynastic Egypt (Caneva 1988, pp. 151–226; Edwards 1996; Geus 1984a, b; Reinold 1987; Sadig 2010; Winchell 2013, pp. 98–100). Settlements appear to have been very light, and the vast majority of the material culture associated with these occupations is found in burials. These occupations appear to represent a dispersed settlement pattern after the apparent abandonment or near abandonment of more concentrated populations along the central Nile Valley that had been associated with the Early Neolithic period at such settlements as Kadero, Shaheinab, and Umm Direiwa (Caneva 1988; Haaland 1987). This period of abandonment and dispersal occurred at around 4150 BC and demarcates the beginning of the Late Neolithic Period (Caneva 1988; Haaland 1987; Sadig 2010).

However, despite winter rainfall cultigens such as wheat and barley being present early on in northern Nubia, i.e., the Dongola Reach, they are unknown further up the Nile into Central Sudan at this time. Furthermore, to date no native, domesticated, African summer rainfall cereals have been found with any of the Neolithic occupations in the central Nile Valley (Sadig 2013, p. 30), with the exception of one possible domesticated-type spikelet reported by Stemler (1990a, b) from Umm Dereiwa.

### Butana and Shaqadud Cave

By contrast, the Central Sudan’s vast triangle of grasslands known as the Butana that stretches across the Sahel between the Nile and Atbara Rivers (Fig. 2) and falls within the uppermost summer rainfall zone. It would have been a prime habitat for hunter-gatherers and pastoralists to exploit natural stands of summer rainfall panicoid cereals. Overall, the southwestern Butana Late Neolithic sites appear to be affiliated to earlier Khartoum Neolithic sites in the central Nile Valley (such as Kadero and Geili). Archaeozoological remains from sites such as Sheikh el Amin contain nearly 90% livestock, but populations still hunted some wild game and subsisted on stands of wild savanna grasses, as groups did along the central Nile Valley further to the north (Fernández et al. 2003, p. 263). Limited archaeobotanical evidence indicates domesticated sorghum only later at Meroitic sites in the Nile, such as Meroe and Hamadab (Fuller 2014), and further south at Jebel Tomat near the White Nile (Clark and Stemler 1975) and at Abu Geili on the Blue Nile in the first few centuries AD (Fuller 2014). However, “edible wild plant impressions” have been recovered from Sheikh el Amin ceramics dating to this period (Abdel-Magid 2003, Table 1; Fernández et al. 2003, p. 263), presumably representing continued use of savanna grasses.

Surprisingly, the northern portion of the Butana appears not to have been as heavily occupied during late prehistoric times as it was in later Meroitic times, though its vast grasslands would have been attractive for hunter-gatherers and made good pasture for cattle herders and/or agro-pastoralists. This problem could be accounted for by the lack of any systematic archaeological investigations focusing on earlier sites. However, the lack of earlier sites in this part of the Butana could also be due to the highly prevalent and conspicuous Meroitic occupations masking more ephemeral sites of an earlier period. Indeed, the Meroitic state expanded from its center in the Nile Valley to take advantage of the rich pasture lands further to the south and east in the Butana, where sorghum could have been handily cultivated, including both more productive *durra* race as well as the basic *bicolor* race (Fuller 2014, 2015).

One of the few known and well-excavated pre-Meroitic occupations known in the Butana is Shaqadud. The multi-component site of Shaqadud, which consists of an open-air mound and midden deposit (Shaqadud Midden) and a cave site (Shaqadud Cave), lies about 50 km east of the Nile. There was a continuous Khartoum Mesolithic to Khartoum Neolithic occupation (ca. 5500–4000 BC). The Mesolithic is reflected in parts of the open-air midden deposit and in full at S21, a shallow artifact distribution a short distance to the east of the western rim of the box canyon. The Neolithic is reflected in the midden and a later Late Neolithic occupation in the adjacent cave dating ca. 2900 to 1600 BC (Fattovich et al. 1984; Marks et al. 1985; Marks and Mohammed-Ali 1991).

During the occupation of Shaqadud Cave, beginning at ca. 2900 BC after a 1100-year hiatus from the abandonment of the open-air midden site, the environmental conditions in the area continued to be grassland savanna (Marks et al. 1985, pp. 264, 275–276; Winchell 2013, p. 102). The excavators hypothesized that the population may have utilized rainfall-based farming, hunting and gathering, and to a lesser extent, the herding of livestock involving goats and sheep, cattle and perhaps some donkey (Marks et al. 1985; Peters 1991, p. 228). Faunal remains were dominated by savanna game as well as taxa from seasonally wet settings, like semi-aquatic *Pila* snails and terrapin turtles (Peters 1991).

Against this backdrop, it is surprising to us that more attention was not paid by the excavators to the macrobotanical remains, which were briefly described in the preliminary report by Magid (1991). The remains derived throughout the cave’s depositional sequence (Magid 1991, p. 193) identified a considerable amount of possible pearl millet grains (reported as *Pennisetum* sp.) attributed to the mid-level deposits (grouped levels 30–48). A few grains of *Panicum turgidum* were also recovered (in group levels 33–27) and a single grain of sorghum (*Sorghum* sp.) was recovered in the lower mid-levels (grouped levels 45–47) (Magid 1991, p. 193). None of these finds was illustrated and it is unknown where they are archived.

Magid (1991, p. 194) also stated that the archaeological *Pennisetum* grain specimens recovered from Shaqadud Cave were similar in exterior features (i.e., size, shape) to the pearl millet crop as currently cultivated in the Sudan further to the south/southeast. On the sorghum specimen, Magid (1991, p. 195) surmised that the grain was similar to modern wild examples found in the Gezira Plain and eastern Butana. In the final assessment, although Magid believed the inhabitants were cultivating pearl millet (as well as sorghum), he was unsure whether the pearl millet specimens recovered from Shaqadud Cave were truly domesticated (1991, p. 196). Based on the frequencies and stratigraphic positions of the pearl millet grains in the cave deposit, the arrival of this species in this part of the Butana would have been prior to 2500 BC (Magid 1991, p. 194, Table 9-1). However, the description alone and lack of illustrations leave open the possibility that either or both of *Pennisetum* and *Sorghum* were of morphologically wild type, or of some mixed/intermediate population as found at KG 23 or Mahal Taglinos.

As with the earlier occupation at the midden and despite geographical proximity, the cultural orientation (namely seen in the ceramic decorations) of the peoples associated with the Late Neolithic occupation at Shaqadud Cave was not towards contemporary communities situated along the Nile Valley to the west, but with other groups further to the east in the southern Atbai (Marks et al. 1985; Marks and Mohammed-Ali 1991; Winchell 2013). Given the hypothesis that the exploitation of pearl millet began in the West African Sahel at least by the fourth millennium BC (before full domestication and dispersal south into the Tilemsi Valley), then it is possible that *Pennisetum* at Shaqadud Cave could have been among some early cultivars that had spread eastwards across the Sahel at this time.

## Discussion

The landscape across the western portion of the southern Atbai, from the interior steppe to the Atbara River, including the spatial distribution among the large Butana Group settlements and the sheer size of the sites themselves, provides good evidence for increasingly sedentary communities (Grove 2007; Sadr 1988, pp. 92–102). For sorghum, domestication was already underway as seen in the ceramic impressions from KG23 during the fourth millennium BC, indicating that the beginning of cultivation was somewhat earlier in this area (Winchell et al. 2017). Unlike contemporary cultures in the Nile Valley engaged with animal husbandry, it was not until the later part of the Butana Group sequence that domesticated animals, such as cattle, sheep, and goat appeared (Peters 1989). An orientation of Butana Group peoples towards the east may be signified by the acquisition of raw materials, such as granite (for grinding and processing foods), porphyry (polished stone mace-heads) sourced in the Eritrean-Ethiopia highlands and the Red Sea Hills (Mbutu 1991, pp. 406, 425–426), and also Red Sea shells for personal ornaments (Manzo 2017, p. 27).

The subsistence strategy of the Butana Group had developed from hunter-gatherers of the Malawiya Group who may have been exploiting wild stands of sorghum, in the savanna regions of the southern Atbai. No artifactual indicators of cultivation were present at that period, although we might infer the earliest experiments with sorghum cultivation to have begun then, given that sorghum domestication is a slow, ongoing process and domesticated sorghum is present in the successor Butana Group. No chaff-tempered ceramics are associated with either the earlier Malawiya Group or the Malawiya/Butana Transition (4000–3800 BC). The Butana Group had more clear cultivation practices, as shown with the introduction of picks for tilling the soil, and there were increasing frequencies of domestic traits in the later stages of pre-domestication sorghum cultivation (see Winchell et al. 2017 and figures within). These morphological representations of the domestication syndrome are visible among impressions and remains recovered from the seed- and chaff-tempered sherds from Butana Group and include non-shattering rachis, plumper spikelets and seeds, and the presence of threshing and dehusking by-products. As pointed out earlier, domesticated animals only appeared in the later part of the Butana Group occupations, thus indicating that the process of sorghum domestication was not involved with pastoralism but began among hunter-gathers communities. Unlike other pastoralist societies across the Sahel who were practicing a transhumant migratory pattern from one region to the next, populations associated with the Butana Group were probably sedentary, living in large year-round settlements.

The non-pastoralist pattern involved with the domestication of sorghum and associated with the Butana Group is different from the domestication of pearl millet in the western Sahel. There in the Tilemsi Valley of eastern Mali, as early as 2500 BC, it appeared that domestic pearl millet was associated with seasonal pastoralists who had arrived in the Valley. They had brought in domesticated pearl millet from elsewhere, however, perhaps from the north along fringes of southwestern Sahara or along the northern edge of the Sahel, where the beginnings of cultivating pearl millet may have occurred as early as 4000 BC (Manning et al. 2011; Manning and Fuller 2014). It was not until 2000 BC that these groups in the Tilemsi Valley appear to have become more sedentary, living in larger settlements, and routinely cultivating pearl millet that had already become domesticated.

This would have been at the same time as the beginnings of sorghum domestication, by the Late Neolithic Butana Group in the southern Atbai. While the time period for the domestication of both pearl millet and sorghum may have been roughly the same, there is now good evidence that sorghum was independently domesticated in the savannas of the far eastern Sahel (Winchell et al. 2017), while pearl millet may have been domesticated somewhere in the far western Sahel (Manning and Fuller 2014).

It is presumed that the spread of domesticated pearl millet from the western portions of the Sahel would have been rapid, as it shows up in India sometime around 2000–1700 BC. Although we know virtually nothing of the Sahelian zone across Nigeria and Chad, neither early domestic pearl millet nor sorghum are archaeologically known in any part of the Sudanic Sahel west of the Nile Valley, nor in any parts of the Nile at that time. Nevertheless, the archaeological deposits at Shaqadud Cave, 50 km east of the Nile in the Butana region, may prove to have an early cultivated form of pearl millet by as early as 2900 BC. It remains a mystery, however, that Late Neolithic occupations along the Nile within 100 km of Shaqadud Cave at such sites as Kadada, el Geili, and Shaheinab contain neither domesticated sorghum nor pearl millet. What may be of some significance, and as pointed out earlier, is that the material culture at Shaqadud Cave does not appear to relate to the contemporary Late Neolithic occupations in the central Nile Valley, but is more oriented to groups farther to the east in the southern Atbai, such as the later part of the Butana Group and beginning of the Gash Group. Thus, it is equally plausible that the practice of intensive cultivation leading into domestication had spread from the southern Atbai (including the actual movements of people), where it had begun centuries before with sorghum, and moved westward across the Butana grasslands arriving at Shaqadud Cave. In this context inhabitants of Shaqadud Cave could have taken up cultivation of pearl millet introduced from the west along with some sorghum. Indeed, more than 10% of the sherds recovered from the Shaqadud Cave deposits consist of chaff/fiber tempered sherds, giving a good indication that threshing of seed harvests was also occurring there, associated with both pearl millet and sorghum, although archaeobotanical study of this chaff tempering is needed. Later still at Mahal Teglinos at ca. 1850 BC, pearl millet was present alongside sorghum and appears to have still included some wild-type, shattering individuals alongside the domesticated form. This might imply that the early dispersal of pearl millet from the western Sahel took place *before* the crop was fully domesticated in a morphological sense.

In the critical period, sometime after the end of the third millennium BC when native African cereals appear in the Indus Valley and Indian subcontinent, it was the Gash Group center at Mahal Teglinos which had established significant trade relations with Egypt, Lower Nubia, and the center at Kerma in Upper Nubia, as wells other parts of the Horn of Africa and southern Arabia. D’Andrea and Wadge (2011) postulate that around 2000 BC, wild-grass-gathering pastoralists of the eastern Sahelian zone, such as those of the southern Red Sea hills or the Gash Delta region, also began to intensify cultivation of savanna grasses such as sorghum. They later expanded into the Ethiopian highlands, adopting tef (*Eragrostis tef*) for cultivation on the northern margins of those highlands. The data now indicate that these groups were indeed already cultivators of sorghum and pearl millet, but further evidence is needed to assess the adoption of other crops such as tef in more southeastern areas.

Thus, it was probably at Mahal Teglinos that people of the Gash Group were first involved with the transference of native African cereals through the Eritrean-Ethiopian highlands, Red Sea Hills, and across the Red Sea via maritime people to as far afield as India. At the sites of Erkowit, in the Red Sea Hills of the Sudan, less than 50 km from the Red Sea, and Agordat, further east at the foot of the Eritrean-Ethiopian highlands, the ceramics associated with these occupations appear to be affiliated with the Gash and Jebel Mokram Groups (Beldados 2010; Beldados et al. 2007; Brandt et al. 2008) and perhaps earlier with the later part of the Butana Group (Winchell 2013, pp. 143–144). At Agordat, seed impressions from *Sorghum* sp. and *Setaria*, sp. were recovered from eight potsherds (dated on typological grounds to ca. 2500–2050 BC). While these were tentatively identified as wild (Beldados et al. 2007, pp. 6–8), the sample size of examined sherds (8 out of a total of 1469 recovered from Agordat) was very small and did not involve the systematic casting of impressions as per recent protocols (e.g., Manning et al. 2011; Winchell et al. 2017). The final assessment that domesticated varieties did not occur there needs to be taken with some caution. Indeed, the researchers at Agordat surmised that the location of this site was in the most eastern natural habitat in the Sahel for sorghum and that the area in general would have been strategic for the transference of sorghum (presumably domesticated or partly domesticated) across the southern Arabian Peninsula and east into India (Beldados et al. 2007, pp. 8–9).

## Conclusion

On the overall origins and distribution of domestic native African cereals, such as sorghum and pearl millet, the savanna zone of the southern Atbai in the far eastern Sahel appears to have been the place where sorghum was both intensively cultivated and first domesticated. Exploitation and cultivation of wild cereals, inclusive of *S. bicolor* subsp. *verticilliflorum*, has a long history, stretching as far back as the Late Acacus in the central Sahara. The fully domesticated varieties of pearl millet in the Tilemsi Valley of southeastern Mali could indicate that this cereal was domesticated somewhere in the western Sahel but had already spread east to Sudan prior to full domestication. This highlights the importance of re-investigating Shaqadud Cave and its archaeobotanical evidence.

The Butana Group in the far eastern Sahel also shows that pre-domestic cultivation for sorghum, and its subsequent domestication, occurred when populations were sedentary, intensifying their cultivation of this grain during the fourth millennium BC. This implies that some centuries before, cultivation of wild stands of sorghum took place as part of a more generalized hunter-gathering strategy, perhaps in the absence of pastoralism. This is in contrast to the widespread pastoralism across the Sahara in the middle Holocene. The idea that cattle came before crops in African food production (e.g., Marshall and Hildebrand 2002) is not, after all, a universal in ancient Africa. In the central Sahara non-sedentary pastoralists appear to have focused on small-grained millets that were prolific and had “weedy” characteristics including resilience to grazing (Mercuri et al. 2018). In the western Sahara, early pastoralists brought pearl millet into cultivation (Manning and Fuller 2014) and the highly bristley spikes of *Pennisetum* were likely less eaten and damaged by livestock. In contrast, wild *Sorghum* spp. are excellent fodder grasses and thus perhaps less fitting for livestock-focused ecologies. Instead, sedentarizing hunter-gatherers appear to have transitioned to early sedentary sorghum cultivators in the eastern Sahel. Thus, the trajectories to domestication of pearl millet and sorghum differed, despite taking place in broadly similar environmental and palaeoclimatic contexts along the southern margins of the gradually expanding Saharan desert. The Acacus region illustrates a third trajectory into cultivation (Mercuri et al. 2018), which did not, in the long term, contribute to traditional agricultural systems of Africa. Both domesticated cereals, pearl millet and sorghum, appear to have been grown together in the far eastern Sahel, certainly by the start of the second millennium BC (Mahal Teglinos) and possibly even earlier (Shaqadud Cave?), prior to being transferred to India via emerging Arabian Sea maritime connections. Taken together, these lines of evidence highlight a series of dynamic societies and subsistence innovators across the Saharan and Sahelian belt, through southern Arabia and semi-arid India that developed new cultivation economies quite distinct from those of western Asia and the Mediterranean.
